# AI Integration in Medical Imaging Education: A Nationwide Cross‐Sectional Survey

**DOI:** 10.1155/rrp/5775572

**Published:** 2026-07-11

**Authors:** Wuni Abdul-Razak, Mawuli Deku, Eric Akpabli, Messiah Narh Kwame Anudjo

**Affiliations:** ^1^ Department of Radiology and Medical Imaging, Fatima College of Health Sciences, Al Ain, UAE, fchs.ac.ae; ^2^ Department of Medical Imaging and Sonography, University of Cape Coast, Cape Coast, Ghana, ucc.edu.gh; ^3^ Department of Nuclear Safety and Security, University of Ghana, Accra, Ghana, ug.edu.gh; ^4^ Institute of Medical Imaging and Visualisation, School for Allied Health and Exercise Science, Faculty of Health, Environment and Medical Sciences, Bournemouth University, Bournemouth, UK, bournemouth.ac.uk; ^5^ Department of Medical Technologies, School of Allied Health Professions and Pharmacy, Faculty of Medicine and Health Sciences, Keele University, Staffordshire, UK, keele.ac.uk

**Keywords:** artificial intelligence, clinical practice, deep learning, educational curriculum, machine learning, neural networks

## Abstract

**Introduction:**

The integration of artificial intelligence (AI) into the medical imaging (MI) curriculum has been advocated by many international professional bodies. This study aimed to explore a nationwide perspective on AI incorporation in the MI curriculum.

**Methods:**

A quantitative cross‐sectional survey was conducted using a self‐administered questionnaire created using Google Forms. The survey focused on the perceived awareness of AI incorporation into the MI curriculum, AI in MI and its impact on service delivery. Descriptive statistics and chi‐square test between institution and AI curriculum incorporation and perceived adequacy were performed using the Statistical Package for Social Sciences (SPSS) (v.26) and Excel 2019.

**Results:**

A total of 200 respondents participated, with the majority being MI students from the University of Cape Coast (77/200, 38.5%). More than half of respondents (124/200, 62.5%) indicated that AI was incorporated into the curriculum, and 87 (44%) reported receiving instruction on AI. A statistically significant correlation was found between institutional affiliation and the perception of AI incorporation and MI (*p* = 0.001).

**Conclusion:**

Respondents noted the incorporation of AI in the MI curriculum, with variations in the level of awareness of AI integration across the various institutions.

**Implication for Practice:**

The evidence presented should help prioritise faculty tutelage and integrate dedicated AI modules to ensure graduates possess the technical competencies and confidence required for safe adoption of AI in practice.

## 1. Introduction

Artificial intelligence (AI) represents a significant advancement in computing, enabling systems to perform tasks that typically require human intelligence [1–5]. The conceptualisation of AI dates back to the 1940s, when John McCarthy coined the term ‘artificial intelligence’ in 1956 [1, 2, 6]. Although earlier AI models had their flaws, the emergence of deep learning (DL) and machine learning (ML) in the early 2000s, coupled with immense advancements in computational power and data availability, subdued most of the drawbacks, propelling AI into a new age of universal acceptance and application [1, 2, 7]. ML and DL are key components of AI, which are composed of algorithms that allow systems to learn and make decisions, usually through artificial neural networks that mimic the human brain [2, 8].

These developments have significantly impacted the healthcare sector, particularly in technologically driven specialties such as radiology, oncology and pathology [6, 9, 10]. Integrating AI within the field of medical imaging (MI) is not a fledgling discipline; it is a reality in modern healthcare [2, 11–15]. Innovation in this space has also been maximised beyond the traditional boundaries of radiology referral (requesting imaging, scheduling process and image acquisition, interpretation or reporting) [11, 16, 17]. These applications of AI in MI are being deployed in positioning, scan planning, dose management, image quality enhancement and imaging task prioritisation across various modalities, including general radiography, computed tomography (CT), magnetic resonance imaging (MRI) and ultrasonography [6, 8, 9, 18, 19].

Given the advancement and growing application of AI in MI, there is a widely acknowledged consensus to equip both current and future MI workforce with foundational and practical competencies in AI [2, 7, 11, 15]. AI in MI has been represented as a ‘Virtual Assistant’ with whom professionals must learn to interact confidently and safely [11]. Therefore, appropriate education is crucial for MI professionals and students to ensure safe and efficient use of clinical AI applications [7, 11, 20].

International regulatory and professional bodies within radiography have emphasised the importance of ensuring that radiographers are adequately prepared and well‐informed regarding both current and emerging AI technologies [11, 21]. For example, the Medical Radiation Practice Board of Australia (MRPBA) has emphasised the need for MI professionals to be adequately educated and actively involved in the safe and effective implementation of AI technologies within clinical environments [21]. Similarly, the United Kingdom’s (UK) Society of Radiographers (SOR) has published guidance that provides recommendations for AI applications in clinical practice, education, research and stakeholder collaboration, aimed at empowering radiographers to promote person‐centred care while effectively integrating AI technologies [22]. Also, the American Society of Radiologic Technologists (ASRT), through its 2024 consensus committee on the ‘Future of Medical Imaging and Radiation Therapy’, advocated for the integration of AI as a compulsory component of the educational curriculum for radiologic science students, rather than an option [22].

Despite the extensive recognition of AI’s adoption in clinical practice and education, the development of appropriate educational curricula remains in its early stages in low‐ and middle‐income countries, such as Ghana. The increasing relevance of AI in MI has become crucial to assess the current state of preparedness of future professionals. Given the emergence and applications of AI in clinical practice and radiography, it is imperative to ensure that radiography curricula are designed to reflect the current needs. This study is therefore aimed at evaluating the awareness of AI applications in radiography among MI students in Ghana and assessing the extent to which AI concepts and applications are integrated into the current radiography curriculum.

## 2. Methods

### 2.1. Study Design and Study Area

This cross‐sectional study was conducted among clinical‐year MI students enrolled in the five public institutions in Ghana offering accredited MI programmes. Although MI programmes are offered by several public and private institutions across Ghana, the broader target population for this study comprised all MI students in Ghana.

The five institutions included in this study were selected because they are among the longest established public training institutions, admitting relatively large student cohorts annually. They also have well‐structured curricula with active clinical training and student engagement. Consequently, these institutions accounted for a substantial proportion of MI graduates in Ghana and provided an appropriately accessible population for the assessment of students’ awareness of AI integration in MI curricula.

### 2.2. Population and Sample Size

There are a total of 673 registered clinical‐year (third‐ and fourth‐year) radiography students across the selected institutions in this study. The total number of third‐ and fourth‐year students was provided by the heads of the various MI departments in the different institutions. The focus on clinical year students is because they are better positioned to reflect on the perceived relevance, applicability and implications of AI in real‐world clinical settings. Introductory AI‐related content, where present, is typically introduced in preclinical years but is contextualised and applied more explicitly during clinical placements and senior‐level modules. Therefore, third‐ and fourth‐year students are more likely to have been exposed to both pedagogic and applied aspects of AI education.

The required sample size for this study was calculated using the formula proposed by Yamane (1967) [23]. To ensure adequate statistical power, a 95% confidence interval and 5% margin of error were used. This yielded a final required sample size of approximately 251. However, accounting for potential nonresponse and incomplete submissions, a 10% attrition allowance was applied. The adjusted sample size was approximately 276.

### 2.3. Sampling Method

A consecutive sampling strategy was employed to recruit participants. This method was selected for its efficiency in reaching a dispersed group [24], enabling the inclusion of all eligible MI undergraduate students available during the data collection window until the target sample size was achieved.

### 2.4. Data Collection

The research instrument was a structured questionnaire developed by the research team on a blank slate. The questionnaire consisted of closed‐ended questions with two sections: Section A comprised participant demographic information, and Section B comprised awareness of AI integration into academic curriculum, teaching and its applications and impact.

To ensure content validity and clarity, the drafted questionnaire was reviewed by experienced academic radiographers and revised based on their feedback. A pilot study was conducted with four representatives from the participating institutions to test reliability and validity. Responses gathered from the pilot were consistent with the study objectives, thus confirming the reliability of the questionnaire. Minor revisions were made based on this feedback to improve clarity and reduce ambiguity. Internal consistency testing, such as Cronbach’s alpha, was not performed, as the instrument included diverse question types designed to capture perceptions and experiences rather than to measure a single construct.

Data collection was conducted over a defined academic period using an online questionnaire. The questionnaire was hosted in Google Forms for data collection and was administered through social media platforms, including WhatsApp, LinkedIn and Facebook. To reach a broader range of respondents, snowball sampling was permitted. The data collection period lasted for 3 months. We implemented strict eligibility criteria for our study, ensuring that qualified participants were third‐ and fourth‐year MI students in Ghana. A multistep verification process assessed their academic status, and ongoing monitoring ensured that ineligible individuals were promptly addressed. This rigorous approach preserved the integrity of our research findings.

### 2.5. Data Analysis

The estimated sample size of the study was 276; the study received responses from 200 participants. Using the G∗Power software (v3.1.9.7), a sample size (*n*) = 276 and alpha = 0.05 (two‐sided), the study yielded 99% statistical power to detect correlations of at least an effective sample size (*ρ*) = 0.3. Prior to analysis, questionnaire responses were coded. Sociodemographic variables were treated as categorical and Likert‐scale perception items numerically coded from 1 (*strongly disagree*) to 5 (*strongly agree*). Higher mean scores reflected more positive awareness or perceptions of AI education. Data were screened for completeness, and missing responses were isolated.

The survey responses were transferred to Excel 2019 for data cleaning, and descriptive statistics were performed. Afterwards, the data were exported into IBM Statistical Package for Social Sciences (SPSS v26.0) for inferential analysis. Percentages were calculated for categorical factors, such as age, sex and educational institution, and frequency distribution tables were used to present the data. Mean scores were calculated for Likert‐scale items to summarise central response tendencies and enable comparison across perception domains. Associations between categorical variables were assessed using a chi‐square test of independence. This analysis was conducted to explore the potential institutional variability in AI educational exposure. Results are reported using the chi‐square statistic (*χ* ^2^), degrees of freedom (df) and corresponding *p* values. Statistical significance was set at *p* < 0.05.

## 3. Results

The majority of participants (80.5%) were aged between 21 and 25 years. The gender distribution shows a higher number of male students (127/200, 63.5%), with a small fraction (2/200, 1.0%) choosing not to disclose their gender. The majority (38.5%) were from the University of Cape Coast, and the University of Ghana (14/200, 7.0%) was the least represented. The majority (113/200, 56.5%) of the respondents were in their third year (Level 300), while the remaining 87/200, 43.5%, were in their final year (fourth year). Further demographic data can be found in Table 1.

**TABLE 1:** Participants’ demographics.

**Age (years)**	**Frequency (*N*)**	**Percentage (%)**

18–20	26	13.0
21–25	161	80.5
26–30	11	5.5
31 and over	2	1.0
Total	200	100.0
Gender		
Male	127	63.5
Female	71	35.5
Prefer not to say	2	1.0
Total	200	100.0
Institutional Affiliations		
University of Cape Coast	77	38.5
University of Health and Allied Sciences	68	34.0
University of Development Studies	26	13.0
University of Ghana	14	7.0
Kwame Nkrumah University of Science and Technology	15	7.5
Total	200	100.0
What level are you currently in?		
300	113	56.5
400	87	43.5
Total	200	100.0

Table 2 presents participants’ views on integrating AI within their curriculum. A significant proportion of students (*n* = 64, 32.5% strongly agreed; *n* = 60, 30.0% agreed) affirmed that AI should be incorporated into their curriculum. Regarding instructional exposure, 87 students (*n* = 33, 17.0% strongly agreed; *n* = 54, 27.0% agreed) reported that they had received teaching on AI in MI. Opinions regarding the adequacy (AI‐related teaching in my MI programme provides depth and relevance to prepare me for clinical application) of AI education in the various schools varied, with only 58 students (*n* = 19, 9.5% strongly agreed; *n* = 39, 20.0% agreed) believing that AI instruction was adequate in their programmes. Notably, 75 students (37.0%) remained neutral, and a considerable proportion (*n* = 45, 22.5% disagreed; *n* = 22, 11.0% strongly disagreed) indicated inadequacy.

**TABLE 2:** AI integration and teaching in curriculum.

Items	Strongly agree	Agree	Neutral	Disagree	Strongly disagree	Mean	SD
You have AI incorporated in your curriculum	64 (32.5)	60 (30.0)	21 (10.5)	34 (16.5)	21 (10.5)	3.56	1.37
You have received teaching on AI in medical imaging	33 (17.0)	54 (27.0)	41 (20.5)	49 (24.0)	23 (11.5)	3.13	1.28
The AI‐related teaching in my medical imaging programme provides depth and relevance to prepare me for clinical application	19 (9.5)	39 (20.0)	75 (37.0)	45 (22.5)	22 (11.0)	2.94	1.12
Your lecturers are adequately knowledgeable in AI in medical imaging	27 (13.5)	67 (34.0)	79 (39.0)	15 (7.5)	12 (6.0)	3.41	1.01
You are prepared to learn more about AI in medical imaging	98 (49.5)	81 (40.0)	18 (9.0)	3 (1.5)	0 (0.0)	4.37	0.71
Having AI in your curriculum would lead to enhanced learning opportunities, research and innovation, efficient workflow and preparation for industry trends	103 (52.0)	74 (36.5)	18 (9.0)	5 (2.5)	0 (0.0)	4.38	0.75

Table 3 highlights the students’ perceived awareness and understanding of AI applications in MI. Generally, the responses indicated a high level of awareness across multiple domains of clinical relevance. Nearly three‐quarters of the students affirmed AI can reduce radiation levels while maintaining image quality (*n* = 58, 29.0% strongly agreed; *n* = 84, 42% agreed). 72% of students (29.5% strongly agreed and 42.5% agreed) acknowledged that AI has been incorporated into key imaging modalities such as CT, MRI and mammography. Approximately 77.0% of students recognised AI’s effectiveness in detecting pathologies in CT and MRI scans (*n* = 66, 33.5% strongly agreed; *n* = 88, 43.5% agreed). In a similar vein, 81.0% of students (*n* = 68, 34.5% strongly agreed; *n* = 94, 46.5% agreed) demonstrated high awareness of AI aiding in selecting technical factors like kVp and mAs.

**TABLE 3:** Perceived awareness and understanding of AI applications in MI.

	**Strongly agree**	**Agree**	**Neutral**	**Disagree**	**Strongly disagree**	**Mean**	**SD**

AI can help to reduce radiation dose levels while maintaining optimal image quality in medical imaging	58 (29.0)	84 (42.0)	41 (20.5)	6 (3.0)	11 (5.5)	3.86	1.05
You are aware that AI has been incorporated into imaging modalities such as CT, MRI and mammography	58 (29.5)	86 (42.5)	43 (21.5)	9 (4.5)	4 (2.0)	3.93	0.93
AI is applied in the following areas in medical imaging: positioning, radiation dose reduction, pathology identification, patient scheduling, patient screening and examination time.	53 (27.0)	92 (45.5)	49 (24.5)	5 (2.5)	1 (0.5)	3.96	0.81
AI helps in detecting pathologies in CT and MRI scans	66 (33.5)	88 (43.5)	43 (21.5)	3 (1.5)	0 (0.0)	4.09	0.78
AI aids in the selection of technical factors such as kVp and mAs in medical imaging	68 (34.5)	94 (46.5)	31 (15.5)	5 (2.5)	2 (1.0)	4.11	0.83
AI aids in positioning in medical imaging	30 (15.0)	64 (32.5)	68 (33.5)	31 (15.5)	7 (3.5)	3.40	1.03

Table 4 presents perceptions and concerns about the broader impact of AI in clinical imaging. The majority, 84.5%, believed that AI will become more critical in the field of MI in the future. Also, less than half of the students thought that AI could lead to job loss (*n* = 28, 14% strongly agreed; *n* = 50, 25.0% agreed). More than half of MI students had concerns regarding data protection, security and data loss (*n* = 46, 23% strongly agreed; *n* = 79, 39.5% agreed) and the majority of students agreed (*n* = 100, 50.5%) and strongly agreed (*n* = 38, 19%) to being worried about the potential ethical implications of AI in MI.

**TABLE 4:** Perceptions and concerns regarding AI’s impact on clinical imaging.

	Strongly agree	Agree	Neutral	Disagree	Strongly disagree	Mean	S.D
AI will become more important in the field of medical imaging in the future	80 (40.5)	89 (44.0)	27 (13.5)	4 (2.0)	0 (0.0)	4.22	0.76
Incorporating AI into medical imaging would lead to job losses in Ghana	28 (14.0)	50 (25.0)	70 (34.5)	28 (14.5)	24 (12.0)	3.15	1.19
You are concerned about potential ethical implications of AI in medical imaging	38 (19.0)	100 (50.5)	50 (24.5)	10 (5.0)	2 (1.0)	3.81	0.83
The following are some specific concerns associated with AI in medical imaging data: protection, security of patient data and data loss	46 (23.0)	79 (39.5)	64 (32.0)	5 (2.5)	6 (3.0)	3.77	0.93
There are possibilities of errors associated with AI‐induced equipment in imaging	40 (20.0)	90 (44.5)	46 (23.0)	10 (5.5)	14 (7.0)	3.66	1.08
AI would have an overall positive impact on medical imaging.	43 (21.5)	82 (40.5)	50 (25.0)	10 (5.5)	15 (7.5)	3.64	1.11

Table 5 presents the results of chi‐square independent tests, examining the relationship between institutional affiliation and perceptions of AI curriculum incorporation and adequacy.

**TABLE 5:** Chi‐square independent test between institution and AI curriculum incorporation and perceived adequacy.

Variable	Total	You have AI incorporated in your curriculum (%)									The AI‐related teaching in my medical imaging programme is adequate and provides depth and relevance to prepare me for clinical application (%)								
		SA	A	N	D	SD	*χ* ^2^	df	*p* Value	C	SA	A	N	D	SD	*χ* ^2^	df	*p* Value	C
University of Cape Coast	77 (38.5)	12 (6.0)	26 (13.0)	12 (6.0)	17 (8.5)	10 (5.0)	87.4	16	0.000	0.331	10 (5.0)	6 (3.0)	24 (12.0)	27 (13.5)	10 (5.0)	55.18	16	0.000	0.263
University of Health and Allied Sciences	68 (34.0)	40 (20.0)	25 (12.5)	2 (1.0)	0 (0.0)	1 (0.5)					8 (4.0)	28 (14.0)	26 (13.0)	4 (2.0)	2 (1.0)				
University of Development Studies	26 (13.0)	9 (4.5)	6 (3.0)	4 (2.0)	3 (1.5)	4 (2.0)					0 (0.0)	4 (2.0)	12 (6.0)	5 (2.5)	5 (2.5)				
University of Ghana	14 (7.0)	1 (0.5)	3 (1.5)	3 (1.5)	4 (2.0)	3 (1.5)					1 (0.5)	1 (0.5)	6 (3.0)	4 (2.0)	2 (1.0)				
Kwame Nkrumah University of Science and Technology	15 (7.5)	2 (1.0)	0 (0.0)	0 (0.0)	10 (5.0)	3 (1.5)					0 (0.0)	0 (0.0)	7 (3.5)	5 (2.5)	3 (1.5)				
Total	200 (100.0)	64 (32.0)	60 (30.0)	21 (10.5)	34 (17.0)	21 (10.5)					19 (9.5)	39 (19.5)	75 (37.5)	45 (22.5)	22 (11.0)				

*Note:* A = agree, N = neutral, D = disagree, C = Cramer’s V, *X* ^2^= chi‐squared value.

Abbreviations: df, degree of freedom; SA, strongly agree; SD, strongly disagree.

A chi‐square test of independence revealed a statistically significant association between institution and reported incorporation of AI into the curriculum (*χ* ^2^(16) = 87.4, *p* < 0.001), indicating that exposure to AI education differed significantly across training institutions. A significant association was also observed between institution and students’ perceived adequacy of AI education (*χ* ^2^(16) = 55.18, *p* < 0.001), suggesting that perceptions of preparedness varied across institutions.

Overall, these findings demonstrate substantial institutional variability in both the variability and perceived adequacy of AI education among imaging students.

## 4. Discussion

The growing deployment of AI algorithms into MI technologies has necessitated the MI profession to take advantage of the potential of AI within the field [23, 25, 26]. Accordingly, professional bodies across the globe have provided guidance for the integration of AI into radiography training and clinical practice. As technology advances, it is imperative that educational curricula evolve accordingly. As such, this study explored the trainee radiography workforce’s awareness of AI’s integration into their educational curricula and the applications of AI in clinical radiography practice. The findings suggest a generally strong understanding of the integration of AI in MI education. Though variations existed in the MI curricula across the various institutions, there is parity in the level of awareness and knowledge of its clinical applications.

### 4.1. Demographics

The survey returned responses from 200 respondents across the selected institutions, revealing demographic patterns that reflect broader educational trends [27]. The study achieved a response rate of 72.5%, with the majority (63.5%) of respondents being males compared to 35.5% female students, while 1.0% chose not to disclose their gender. The male dominance in this study aligns with a similar study conducted on MI students in Ghana by Ampofo et al. [28] and the general male‐dominance MI profession [29, 30], but contrasts with the female dominance in MI education in developed countries such as the UK [31, 32]. The majority (80.5%) of participants were aged 21–25, which is consistent with typical undergraduate enrolment patterns across Ghana’s tertiary institutions. This demographic characteristic reflects the standard progression through Ghana’s educational system, where students enter university after completing high school, usually around the age of 18–19. University of Cape Coast had the highest attendance rate, with 38.5% of participants. Kwame Nkrumah University of Science and Technology (7.5%) and E (7.0%) had the lowest attendance rates.

### 4.2. AI Integration and Teaching in Curriculum

The findings from this study indicate a progressive shift towards the integration of AI within the MI curriculum, with 62.5% of students acknowledging its inclusion. However, a follow‐up question revealed that only 44% of students had received direct teaching on AI in MI, suggesting that while AI concepts may be present, they are likely ingrained within broader course content rather than delivered through standalone modules. This aligns with recent efforts to modernise allied health curricula in response to technological advancement, but contrasts notably with Ampofo et al. [28], who observed an absence of integration of AI in the undergraduate MI curriculum in Ghana. The observed discrepancy may reflect recent curriculum revisions or the informal introduction of AI topics in existing teaching modules, stressing the need for more comprehensive curriculum frameworks and standardised approaches to AI education across institutions. These findings raise significant considerations for educators and policymakers to ensure that MI graduates are adequately equipped for the AI‐driven clinical imaging environment. This reflects the rapid revolution of MI education in response to global AI trends.

Akudjedu et al. [33] reported radiographers’ initial reluctance in adopting AI, possibly due to hesitation in accepting the changes AI might bring to workflows, processes and services. This resistance may have led to a lack of awareness and visibility of AI initiatives, including educational provisions promoting AI literacy. Currently, AI’s growing importance in MI has likely prompted various institutions to integrate AI content into their curricula. Also, students perceived the adequacy of AI education as moderate, suggesting that while AI may be incorporated in educational curricula, there is more room to improve its depth and comprehensiveness.

Statistically significant correlation existed between institutional affiliations and the perception of adequacy of AI education.

The significant associations observed between institution and both AI curriculum incorporation and perceived adequacy highlight uneven integration of AI education across training institutions. This suggests that students’ exposure to AI competencies may depend largely on institutional curricular priorities and available educational resources. University of Health and Allied Sciences showed notably higher levels of student agreement regarding AI incorporation, with 20.0% strongly agreeing and 12.5% agreeing. University of Health and Allied Sciences has recently incorporated an AI‐powered simulation lab as part of its efforts to enhance AI education in MI [34]. This could likely impact the students’ positive agreement to the incorporation of AI. In contrast, Kwame Nkrumah University of Science and Technology had a higher percentage (6.5%) of students disagreeing with AI incorporation. These institutional differences likely reflect varying approaches to curriculum development, faculty expertise in AI technology and institutional priorities regarding emerging technology in MI education. These findings highlight the need for coordinated national curriculum guidance to ensure equitable AI competency development among future MI professionals.

### 4.3. Awareness and Application of AI in MI

Table 3 details the perceived awareness of MI students in AI applications. MI students demonstrated high awareness of AI’s potential in reducing radiation doses while maintaining image quality, with 71.0% agreeing (29.0% strongly agreed, 42.0% agreed). Also, high perception was noted regarding AI’s assistance in selecting technical factors like kVp and mAs, with 81.0% agreeing. This awareness aligns with current studies showing AI’s important role in radiation dose optimisation through improved image reconstruction algorithms and protocol optimisation [12, 14, 35, 36].

Furthermore, students demonstrated a high level of awareness regarding the integration of AI into various MI modalities. 72.0% acknowledged the presence of AI in CT, MRI and mammography. This high awareness reflects the increasing visibility of AI applications across multiple imaging platforms in clinical practice [2]. In recent years, AI has been deployed in the detection of specific pathologies. Through the introduction of computer‐aided detection (CAD), AI has been utilised to reduce patients’ turnaround times, enhance diagnoses and inform treatment planning [37]. In this instance, MI students reported significant awareness of AI’s role in detecting pathologies in MI. 77.0% agreed to this assertion. This recognition is consistent with existing literature demonstrating AI’s effectiveness in medical image analysis and diagnosis [38, 39].

### 4.4. Perceptions and Concerns Regarding AI’s Impact

MI students generally reported varying concerns regarding AI in MI, with 39.0% of students expressing worry about potential job losses due to AI (Table 4). This mirrors international findings where medical students and early career professionals expressed higher levels of anxiety about AI’s impact on employment [40]. In a similar study, 43% of medical students believed that AI threatened their job security compared to 6% of practicing radiologists [41]. This suggests that education and exposure to AI applications may help alleviate unfounded concerns about job displacement [42]. Concerns were also raised about data protection and security issues. MI students expressed significant concerns regarding data protection, security and data loss, with 62.5% agreeing that these were important issues. This reflects global discussion on AI ethics, privacy and security in healthcare applications [43]. Also in our study, an overwhelming majority (84.5%) of the students believed AI would become increasingly important in MI, indicating strong recognition of AI’s transformative potential. This finding is supported by existing literature [41].

### 4.5. Recommendations

Based on the findings of this study, the following recommendations can be adopted.

1. Enhance faculty expertise in AI training.
2. Continuous curriculum review and evolution to reflect currency and relevance.
3. Extending the current study to include other African countries in response to global technological advancements.
4. Regulatory authorities must make proficiency in AI in MI a compulsory graduate competency and enforce this through the training institutions.

### 4.6. Limitations of the Study

The data collection approach is a limitation as self‐reported data introduce bias, as participants’ responses might not reflect the reality on the ground. Besides, using closed‐ended questions impedes the gathering of rich and in‐depth responses. Also, given that the survey was shared via social media, it meant that potentially suitable participants who are not on any of these platforms cannot access the survey to respond to it. The achieved sample size (200) fell short of the calculated minimum (276); notwithstanding this, a post hoc statistical power determination using G∗Power software (v3.1.9.7) yielded a 99% power. Additionally, the study focused exclusively on clinical‐year students because they are closest to clinical practice and most likely to have been exposed to AI‐related content and clinical applications. As a result, the findings cannot be generalised to preclinical students, whose exposure to and perceptions of AI may differ.

## 5. Conclusion

A general positive awareness of AI integration into MI curriculum was observed among Ghanaian students as opposed to an earlier study by Ampofo et al. [28]. Despite variations in AI incorporation across institutions, there is an apparent demand for more robust AI‐related education in MI. Participants also showed high awareness of AI’s potential in reducing radiation doses, maintaining image quality and assisting in detecting pathologies. Ethical implications and job losses were concerns that MI students raised.

## Author Contributions

Wuni Abdul‐Razak, Mawuli Deku, Eric Akpabli and Messiah Narh Kwame Anudjo: conceptualisation, methodology and software.

Wuni Abdul‐Razak, Mawuli Deku, Eric Akpabli and Messiah Narh Kwame Anudjo: data curation, writing–original draft preparation.

Wuni Abdul‐Razak, Mawuli Deku, Eric Akpabli and Messiah Narh Kwame Anudjo: visualisation and investigation.

Wuni Abdul‐Razak: supervision.

Wuni Abdul‐Razak, Mawuli Deku, Eric Akpabli and Messiah Narh Kwame Anudjo: software and validation.

Wuni Abdul‐Razak, Mawuli Deku, Eric Akpabli and Messiah Narh Kwame Anudjo: writing–reviewing and editing.

## Funding

This study did not receive any specific grant from funding agencies in the public, commercial, or not‐for‐profit sectors.

## Ethics Statement

The study received ethical clearance from the Institutional Review Board (IRB) of the University of Cape Coast (Ethics Approval ID: UCC‐319/2024). This study was carried out in accordance with the ethical principles of the 1964 Declaration of Helsinki and its later amendments.

## Consent

Participants were provided with a detailed participant information form that explained the study’s purpose, participants’ role and their rights, emphasising that their participation is voluntary and that their data would be held confidential. All participants signed an electronic informed consent form before accessing the survey. To protect participants’ privacy, all data were anonymised and securely stored in the lead researcher’s institutional Microsoft OneDrive.

The IRB thoroughly reviewed the study to ensure compliance with institutional policies and ethical standards, addressing and resolving any ethical concerns that were raised. Written informed consent was obtained for anonymised patient information to be published in this article.

## Conflicts of Interest

The authors declare no conflicts of interest.

## Data Availability

Data required for this study may be made available by the authors upon reasonable request.

## References

[1] Kaul V. , Enslin S. , and Gross S. A. , History of Artificial Intelligence in Medicine, Gastrointestinal Endoscopy. (2020) 92, no. 4, 807–812, 10.1016/j.gie.2020.06.040. 32565184

[2] Barragán-Montero A. , Javaid U. , Valdés G. et al., Artificial Intelligence and Machine Learning for Medical Imaging: A Technology Review, Physica Medica. (2021) 83, 242–256, 10.1016/j.ejmp.2021.04.016. 33979715 PMC8184621

[3] Loi S. J. , Ng W. , Lai C. , and Chua E. C.-P. , Artificial Intelligence Education in Medical Imaging: A Scoping Review, Journal of Medical Imaging and Radiation Sciences. (2024) 56, no. 2, 10.1016/j.jmir.2024.101798. 39718290

[4] Botwe B. O. , Akudjedu T. N. , Antwi W. K. et al., The Integration of Artificial Intelligence in Medical Imaging Practice: Perspectives of African Radiographers, Radiography. (2021) 27, no. 3, 861–866, 10.1016/j.radi.2021.01.008. 33622574

[5] Briganti G. and Moine O. L. , Artificial Intelligence in Medicine: Today and Tomorrow, Frontiers of Medicine. (2020) 7, no. 27, 10.3389/fmed.2020.00027. PMC7012990 32118012

[6] Drukker L. , Noble J. A. , and Papageorghiou A. T. , Introduction to Artificial Intelligence in Ultrasound Imaging in Obstetrics and Gynecology, Ultrasound in Obstetrics and Gynecology. (2020) 56, no. 4, 498–505, 10.1002/uog.22122. 32530098 PMC7702141

[7] van de Venter R. , Skelton E. , Matthew J. et al., Artificial Intelligence Education for Radiographers, an Evaluation of a UK Postgraduate Educational Intervention Using Participatory Action Research: A Pilot Study, Insights Into Imaging. (2023) 14, no. 1, 10.1186/s13244-023-01372-2. PMC9897152 36735172

[8] Chassagnon G. , Vakalopoulou M. , Paragios N. , and Revel M.-P. , Artificial Intelligence Applications for Thoracic Imaging, European Journal of Radiology. (2020) 123, 10.1016/j.ejrad.2019.108774. 31841881

[9] Pinto-Coelho L. , How Artificial Intelligence is Shaping Medical Imaging Technology: A Survey of Innovations and Applications, Bioengineering. (2023) 10, no. 12, 10.3390/bioengineering10121435. PMC10740686 38136026

[10] Kulkarni S. , Seneviratne N. , Baig M. S. , and Khan A. H. A. , Artificial Intelligence in Medicine: where are We Now?, Academic Radiology. (2019) 27, no. 1, 62–70, 10.1016/j.acra.2019.10.001. 31636002

[11] Crotty E. , Singh A. , Neligan N. , Chamunyonga C. , and Edwards C. , Artificial Intelligence in Medical Imaging Education: Recommendations for Undergraduate Curriculum Development, Radiography. (2024) 30, 67–73, 10.1016/j.radi.2024.10.008. 39454460

[12] Hardy M. and Harvey H. , Artificial Intelligence in Diagnostic Imaging: Impact on the Radiography Profession, British Journal of Radiology. (2019) 93, no. 1108, 10.1259/bjr.20190840. PMC7362930 31821024

[13] Gore J. C. , Artificial Intelligence in Medical Imaging, Magnetic Resonance Imaging. (2019) 68, no. A1-A4, A1–A4, 10.1016/j.mri.2019.12.006. 31857130

[14] Lewis S. J. , Gandomkar Z. , and Brennan P. C. , Artificial Intelligence in Medical Imaging Practice: Looking to the Future, Journal of Medical Radiation Sciences. (2019) 66, no. 4, 292–295, 10.1002/jmrs.369. 31709775 PMC6920680

[15] Donkor A. , Kumi D. , Amponsah E. , and Della Atuwo-Ampoh V. , Principles for Enhancing Trust in Artificial Intelligence Systems Among Medical Imaging Professionals in Ghana: A Nationwide cross-sectional Study, Radiography. (2025) 31, no. 3, 10.1016/j.radi.2025.102953. 40228323

[16] Anzai Y. , Heilbrun M. E. , Haas D. et al., Dissecting Costs of CT Study: Application of TDABC (Time-Driven Activity-Based Costing) in a Tertiary Academic Center, Academic Radiology. (2017) 24, no. 2, 200–208, 10.1016/j.acra.2016.11.001. 27988200

[17] Lee H. W. , Jin K. N. , Oh S. et al., Artificial Intelligence Solution for Chest Radiographs in Respiratory Outpatient Clinics: Multicenter Prospective Randomized Clinical Trial, Annals of the American Thoracic Society. (2023) 20, no. 5, 660–667, 10.1513/annalsats.202206-481oc. 36508316

[18] Wang S. , Cao G. , Wang Y. et al., Review and Prospect: Artificial Intelligence in Advanced Medical Imaging, Frontiers in Radiology. (2021) 1, 10.3389/fradi.2021.781868. PMC10365109 37492170

[19] Najjar R. , Redefining Radiology: A Review of Artificial Intelligence Integration in Medical Imaging, Diagnostics. (2023) 13, no. 17, 10.3390/diagnostics13172760. PMC10487271 37685300

[20] Tejani A. S. , Elhalawani H. , Moy L. , Kohli M. , and Kahn C. E. , Artificial Intelligence and Radiology Education, Radiology: Artificial Intelligence. (2022) 5, no. 1, 10.1148/ryai.220084. PMC9885376 36721409

[21] Murphy A. and Liszewski B. , Artificial Intelligence and the Medical Radiation Profession: How our Advocacy Must Inform Future Practice, Journal of Medical Imaging and Radiation Sciences. (2019) 50, no. 4, S15–S19, 10.1016/j.jmir.2019.09.001. 31611013

[22] Malamateniou C. , McFadden S. , McQuinlan Y. et al., Artificial Intelligence: Guidance for Clinical Imaging and Therapeutic Radiography Professionals, a Summary by the Society of Radiographers AI Working Group, Radiography. (2021) 27, no. 4, 1192–1202, 10.1016/j.radi.2021.07.028. 34420888

[23] Carlson R. V. , Boyd K. M. , and Webb D. J. , The Revision of the Declaration of Helsinki: Past, Present and Future, British Journal of Clinical Pharmacology. (2004) 57, no. 6, 695–713, 10.1111/j.1365-2125.2004.02103.x. 15151515 PMC1884510

[24] Setia M. S. , Methodology Series Module 3: Cross-Sectional Studies, Indian Journal of Dermatology. (2016) 61, no. 3, 261–264, 10.4103/0019-5154.182410. 27293245 PMC4885177

[25] Coakley S. , Young R. , Moore N. et al., Radiographers’ Knowledge, Attitudes and Expectations of Artificial Intelligence in Medical Imaging, Radiography. (2022) 28, no. 4, 943–948, 10.1016/j.radi.2022.06.020. 35839662

[26] Amegadzie J. K. , Kanubala D. D. , Cobbina K. A. , and Acquaye C. , State and Future Prospects of Artificial Intelligence (AI) in Ghana, 2021, 10.22624/aims/isteams-2021/v27p1.

[27] Yang J. , Blount Y. , and Amrollahi A. , Artificial Intelligence Adoption in a Professional Service Industry: A Multiple Case Study, Technological Forecasting and Social Change. (2024) 201, 10.1016/j.techfore.2024.123251.

[28] Ago J. L. , Anim-Sampong S. , Loyal et al., Insights Into Radiography Education in Ghana: Document Analysis of Curricula Contents and Perspectives of Selected Stakeholders, Research Square (Research Square). (2025) 10.21203/rs.3.rs-5759313/v1.

[29] Ampofo J. W. , Emery C. V. , and Ofori I. N. , Assessing the Level of Understanding (Knowledge) and Awareness of Diagnostic Imaging Students in Ghana on Artificial Intelligence and its Applications in Medical Imaging, Radiology Research and Practice. (2023) 2023, 1–9, 10.1155/2023/4704342. PMC10287516 37362195

[30] Luntsi G. , Radiography Profession: Regulation, Practice and Challenges in Northern Nigeria, ResearchGate. (2015) 29, no. 1, 1–8, https://www.researchgate.net/publication/320012134_Radiography_Profession_Regulation_Practice_and_Challenges_in_Northern_Nigeria.

[31] Anim-Sampong S. and Nkansah J. , Women in Radiography Practice in Ghana: Motivating and Demotivating Factors, 2018, https://www.researchgate.net/publication/324773005. 29691344

[32] Akudjedu T. N. , Torre S. , Khine R. , Katsifarakis D. , Newman D. , and Malamateniou C. , Knowledge, Perceptions, and Expectations of Artificial Intelligence in Radiography Practice: A Global Radiography Workforce Survey, Journal of Medical Imaging and Radiation Sciences. (2022) 0, no. 0, 104–116, 10.1016/j.jmir.2022.11.016. 36535859

[33] Akudjedu T. N. , Lawal O. , Sharma M. et al., Impact of the COVID-19 Pandemic on Radiography Practice: Findings From a UK Radiography Workforce Survey, BJR|Open. (2020) 2, no. 1, 10.1259/bjro.20200023. PMC7583354 33178980

[34] Malamateniou C. , Knapp K. M. , Pergola M. , Woznitza N. , and Hardy M. , Artificial Intelligence in Radiography: Where are we now and what Does the Future Hold?, Radiography. (2021) 27, no. 1, S58–S62, 10.1016/j.radi.2021.07.015. 34380589

[35] University of Health and Allied Sciences , Did You Know? University of Health and Allied Sciences Has the Biggest Simulation Center in Africa, 2022.

[36] Bani-Ahmad M. , England A. , McLaughlin L. , Hadi Y. H. , and McEntee M. , Potential of Artificial Intelligence for Radiation Dose Reduction in Computed Tomography—A Scoping Review, Radiography. (2025) 31, no. 4, 10.1016/j.radi.2025.102968. 40339443

[37] McCollough C. H. and Leng S. , Use of Artificial Intelligence in Computed Tomography Dose Optimisation, Annals of the ICRP. (2020) 49, no. 1, 113–125, 10.1177/0146645320940827. 32870019

[38] Barreiro-Ares A. , Morales-Santiago A. , Sendra-Portero F. , and Souto-Bayarri M. , Impact of the Rise of Artificial Intelligence in Radiology: What do Students Think?, International Journal of Environmental Research and Public Health. (2023) 20, no. 2, 10.3390/ijerph20021589. PMC9867061 36674348

[39] Samala R. K. , Drukker K. , Shukla-Dave A. et al., AI and Machine Learning in Medical Imaging: Key Points From Development to Translation, Deleted Journal. (2024) 1, no. 1, 10.1093/bjrai/ubae006. PMC11140849 38828430

[40] Sallam M. , Al-Mahzoum K. , Almutairi Y. M. et al., Anxiety Among Medical Students Regarding Generative Artificial Intelligence Models: A Pilot Descriptive Study, International Medical Education. (2024) 3, no. 4, 406–425, 10.3390/ime3040031.

[41] Yee K. M. , Medical Students Concerned About AI’s Impact on Radiology, AuntMinnie. (2023) https://www.auntminnie.com/imaging-informatics/artificial-intelligence/article/15633684/medical-students-concerned-about-ais-impact-on-radiology.

[42] Arif W. , Radiologic Technology Students’ Perceptions on Adoption of Artificial Intelligence Technology in Radiology, International Journal of General Medicine. (2024) 17, 3129–3136, 10.2147/ijgm.s465944. 39049835 PMC11268710

[43] Koçak B. , Ponsiglione A. , Stanzione A. et al., Bias in Artificial Intelligence for Medical Imaging: Fundamentals, Detection, Avoidance, Mitigation, Challenges, Ethics, and Prospects, Diagnostic and interventional radiology. (2024) 31, no. 2, 10.4274/dir.2024.242854. PMC11880872 38953330

